# Expanding the Clinical Spectrum of *CRB1*-Retinopathies: A Novel Genotype–Phenotype Correlation with Macular Dystrophy and Elevated Intraocular Pressure

**DOI:** 10.3390/ijms26072836

**Published:** 2025-03-21

**Authors:** Ana Catalina Rodriguez-Martinez, Oliver R. Marmoy, Katrina L. Prise, Robert H. Henderson, Dorothy A. Thompson, Mariya Moosajee

**Affiliations:** 1 Clinical and Academic Department of Ophthalmology, Great Ormond Street Hospital for Children NHS Foundation Trust, London WC1N 1LE, UK; ana.rodriguezmartinez@nhs.net (A.C.R.-M.); oliver.marmoy@gosh.nhs.uk (O.R.M.); katrina.prise@gosh.nhs.uk (K.L.P.); robert.henderson@gosh.nhs.uk (R.H.H.); dorothy.thompson@gosh.nhs.uk (D.A.T.); 2 UCL Institute of Ophthalmology, London EC1V 9EL, UK; 3 Moorfields Eye Hospital NHS Foundation Trust, London EC1V 2PD, UK; 4 UCL-GOSH Institute of Child Health, London WC1N 1EH, UK; 5 The Francis Crick Institute, London NW1 1AT, UK

**Keywords:** *CRB1*, crumbs homologue 1, macular dystrophy, cystoid macular oedema, ERG, ON-pathway

## Abstract

Biallelic pathogenic variants in the *CRB1* gene are associated with severe retinal dystrophies, including early onset severe retinal dystrophy/Leber congenital amaurosis (EOSRD/LCA), retinitis pigmentosa (RP), cone–rod dystrophy (CORD), and macular dystrophy (MD). Despite growing research, scant genotype–phenotype correlations have been established. Here, we present two cases involving individuals that presented with cystoid macular oedema and high intraocular pressure, which were later diagnosed as *CRB1*-MD, demonstrating a mild and stable phenotype. Two unrelated patients of African heritage were included, a 7-year-old female (case 1) and a 25-year-old female (case 2), both presenting with ocular hypertension and cystoid macular oedema. Case 2 had a history of bilateral plateau iris, treated with laser iridotomy. Baseline visual acuity for case 1 was 0.66 logMAR in the right eye and 0.54 logMAR in the left eye. For case 2, visual acuity was recorded as 0.30 logMAR in both eyes. Genetic testing confirmed a homozygous c.2506C>A p.(Pro836Thr) variant in the *CRB1* gene in both cases. Longitudinal follow-up over seven years revealed stable visual acuity, improvement of cystoid macular oedema, and effective intraocular pressure control with topical ocular hypotensive therapy. This study establishes a novel genotype–phenotype correlation between the c.2506C>A p.(Pro836Thr) variant and MD, suggesting a mild, stable disease course in homozygous cases. The findings also highlight a potential association of this variant with elevated IOP, expanding the clinical spectrum of *CRB1*-related ocular conditions. Early genetic diagnosis and regular ophthalmic monitoring are essential to optimise management and identify therapeutic opportunities in patients with mild *CRB1*-related phenotypes.

## 1. Introduction

The Crumbs cell polarity complex component 1 gene (*CRB1*, OMIM #604210), located on chromosome 1q31.3, encodes three protein isoforms, CRB1-A, B, and C. The canonical CRB1-A is a single-pass transmembrane protein consisting of an extracellular domain with 19 epidermal growth factor (EGF)-like repeats and 3 laminin G-like domains [1,2,3]. The CRB1-B, comprising 1003 amino acids, shares structural similarity with CRB1-A in the extracellular domains but possesses distinctive 5′ and 3′ domains [4,5,6]. CRB1-C, comprising 754 amino acids, lacks transmembrane and intracellular domains [7,8]. Within the retina, CRB1 is localised in the subapical region above the adherens junctions between photoreceptors and Müller glial cells (MGCs) and photoreceptors [4]. Müller cells exclusively express the canonical CRB1-A, whereas photoreceptors express mainly CRB1-B [4], and CRB1-C function remains uncertain [7,8].

CRB1 plays a key role in regulating essential cellular processes, including apical–basal polarity, outer limiting membrane (OLM) integrity, cell–cell adhesion, and signalling pathways [6]. It is particularly important for retinal development and long-term maintenance, contributing to the stability of zonula adherens junctions at the OLM [9]. Within these adherence junctions, CRB1 interacts with PALS1 (also known as MPP5), PALS1-associated tight junction protein (PATJ), and MUPP1 [10]. Together, these proteins coordinate signalling pathways that influence cell proliferation, cell fate, and the formation of epithelial adherence junctions [10]. Retinal organoids derived from RP-*CRB1* patients homozygous for c.3122T>C p.(Met1041Thr) have shown significantly reduced photoreceptor nuclei and ONL thickness compared to isogenic controls [11].

Biallelic pathogenic variants in the *CRB1* gene result in a diverse range of retinopathies which cause severe retinal degeneration and visual impairment from an early age [12]. The most frequent phenotype observed is Leber congenital amaurosis (OMIM #613935, LCA8), accounting for 7–17% of cases, followed by autosomal recessive retinitis pigmentosa (OMIM #600105, RP12), representing 3–9% of cases. Less common forms include cone–rod dystrophies (CORD). accounting for 6.5% of CORD cases [12], and macular dystrophy (MD), the exact prevalence of which is unknown. Common features seen in these conditions are nummular pigmentation, fine yellow punctate deposits, preserved para-arteriolar retinal pigment epithelium (PPRPE), a coarse, thickened retina, and foveal hypoplasia seen on Optical Coherence Tomography (OCT) scans [13]. It may also be accompanied by peripheral exudative retinal capillary telangiectasia, resembling Coats disease vasculopathy [14,15]. Within the MD phenotype, observed findings are localised to the macula, intriguingly sparing the foveola [16]. It has an unusual degeneration pattern, affecting the superior, inferior, and nasal retina close to the optic nerve; one that is shared with retinopathies caused by the *ADAM9* and *CDH3* genes [16].

To date, limited genotype–phenotype correlations have been established in *CRB1* retinopathies. However, frameshift, nonsense, and splicing variants have been associated with increased disease severity [17]. Furthermore, recent advances in understanding CRB1 isoform expression in retinal cells have provided further insights, particularly for the MD phenotype. An example is the in-frame deletion c.498_506del p.(Ile167_Gly169del), which has been consistently linked to MD and observed in patients with relatively milder forms of generalised retinal disease, suggesting that this in-frame deletion may act as a hypomorphic allele [16]. This variant, located in exon 2, affects CRB1-A while sparing CRB1-B, destabilising the local folding and orientation of two EGF-like modules, and compromising the structural organisation of the CRB1 protein [4]. The preserved function of CRB1-B likely contributes to a less severe clinical presentation [4]. Beyond this well-established genotype–phenotype correlation for MD, no other variants have been directly associated with this presentation. This study introduces two cases of unexplained cystoid macular oedema and elevated intraocular pressure, later identified to carry the homozygous c.2506C>A; p.(Pro836Thr) mutation in the *CRB1* gene. These findings suggest a novel genotype–phenotype correlation with MD and potentially implicate the *CRB1* variant in raised intraocular pressure (IOP) and increased risk of glaucoma.

## 2. Results

### 2.1. Case 1

**Figure 1:**
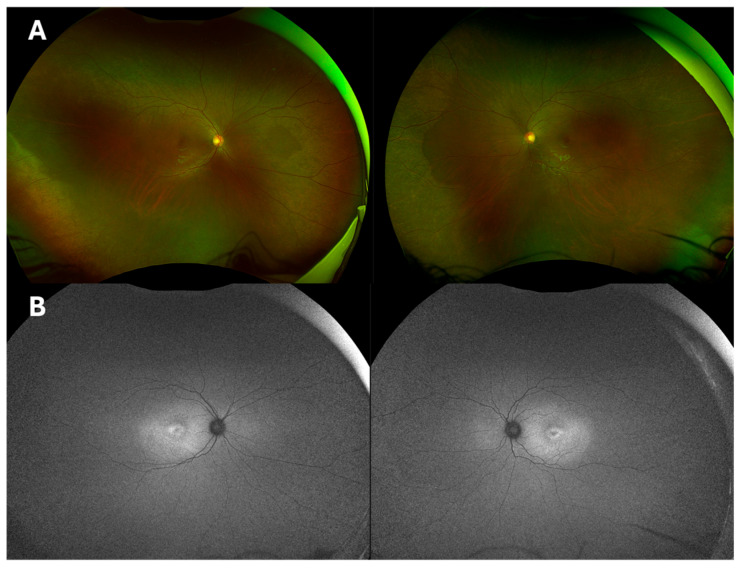
(**A**). Widefield colour fundus photographs of Case 1 showing normal peripheral retinal appearance with no vessel narrowing, no bone spicules, and no nummular pigmentation. (**B**). Corresponding fundus autofluorescence (FAF) depicting parafoveal hypo autofluorescence, surrounded by hyperfluorescent patterns.

A seven-year-old female of African heritage (Sierra Leone) was referred to Great Ormond Street Hospital (London, UK) for bilateral reduced visual acuity and foveal retinoschisis, noted by her local optician. No ocular conditions or family history of ocular conditions were reported. Her general history was unremarkable except for an accessory ulnar digit of the left hand being surgically removed at the age of five. She was prescribed glasses by an optometrist around the age of 5 years to correct a myopic astigmatism. Upon examination at 7 years of age, her best-corrected visual acuity (BCVA) was 0.66 LogMAR from her right eye and 0.54 LogMAR from her left eye. The Pelli-Robson contrast sensitivity test resulted in values of 1.1 in each eye, and Ishihara colour vision testing indicated full colour vision. Goldman applanation tonometry revealed significantly elevated IOP (RE: 29 mmHg, LE: 44 mmHg) alongside thin central corneal thicknesses (RE: 508 μm, LE: 504 μm). Although glaucoma was excluded, with no features on OCT, the patient was diagnosed with ocular hypertension and commenced on brinzolamide and latanoprost. Fundoscopy depicted peripheral retina within normal limits (Figure 1A). Fundus autofluorescence (FAF) demonstrated slightly greater autofluorescence signal around the posterior pole, with a bulls-eye-type pattern of hypo-autofluorescence around the maculae (Figure 1B). Baseline OCT imaging revealed large intraretinal foveal cysts, with subtler schitic-type intraretinal cysts within the inner nuclear layer (INL), increased central retinal thickness (CRT), and central ellipsoid zone disruption with slightly coarse lamination. Whole exome sequencing identified the homozygous c.2506C>A; p.(Pro836Thr) pathogenic variant in the *CRB1* gene, with both parents confirmed as heterozygous carriers through segregation analysis.

**Figure 2:**
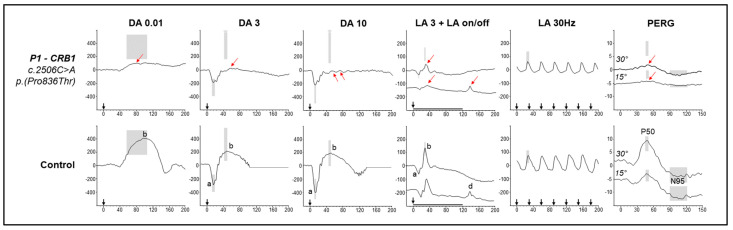
Full-field electroretinography (ERG) and pattern ERG for case 1. The top panels illustrate the patient responses and the bottom panel examples from a healthy control patient. Laboratory reference limits are illustrated by the grey boxes. Black arrows marked on the X axis indicate stimulus timing. The dark adapted (DA) ffERGs showed reduced b-wave amplitude to all stimuli, (single red arrows), but with a-wave amplitude within reference range, albeit toward the lower reference limit. The DA 10 ERG showed low frequency slow oscillations following the b-wave (two red arrows). The light adapted (LA) ffERG showed a reduced b-wave amplitude with borderline peak-time. The prolonged (120 ms) On–Off LA ffERG had a broad b-wave on-response and simplified d-wave off-response complex. The 30 Hz flicker ERG was within reference range, leaning toward lower reference limits. The PERG P50 amplitude was reduced in both a large (30°) and standard (15°) field, with a preserved N95:P50 ratio.

Electrodiagnostic testing was performed, including full-field electroretinogram (ERG), pattern ERG, and pattern VEP, incorporating ISCEV standards [18,19,20]. The full-field ERGs revealed mildly reduced b-wave amplitudes, indicating mild generalised inner retinal dysfunction affecting both rod and cone pathways, with preservation of photoreceptor function indicated from the a-wave amplitude and peak-time. Notably, the morphology of the DA3 scotopic ERG b-wave was observed to have a later low-frequency oscillation, and photopic ERGs exhibited a broad ON b-wave and normal OFF response (Figure 2). Pattern ERG P50 amplitudes were reduced within the central 15° and 30° degree fields, indicating macular cone dysfunction affecting the central 30°, with relative preservation of the N95:P50 ratio. This was performed on two occasions (age 7 and 10) and remained stable across visits. Pattern VEPs demonstrated slightly prolonged P100 peak times and became attenuated to the smallest check widths (6′).

Upon her most recent follow-up scans (7-year follow-up), improvement of intraretinal cysts and central retinal thickness (CRT) were noted. Minimal disruption of the ellipsoid zone was noted. IOP control (RE: 25 mmHg, LE: 25 mmHg) was achieved with topical Brinzolamide BD and Latanoprost once daily. BCVA, on her most recent visit, was a stable 0.50 LogMAR on her right eye and 0.60 LogMAR on her left eye (Table 1).

### 2.2. Case 2

**Figure 3:**
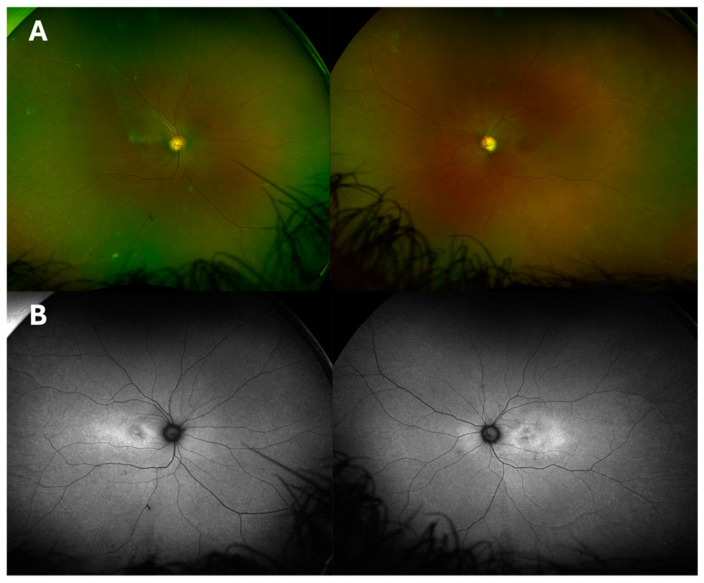
(**A**). Widefield colour fundus photographs of Case 2, depicting normal peripheral retinal appearance with fine yellow punctate deposits and blunt macular reflex on either eye; optic nerve showed a C/D of 0.5 on the right eye and 0.7 on the left eye (**B**). Corresponding fundus autofluorescence (FAF) depicting parafoveal hypo autofluorescence surrounded by hyperfluorescent patterns, more pronounced inferiorly (LE > RE).

**Figure 4:**
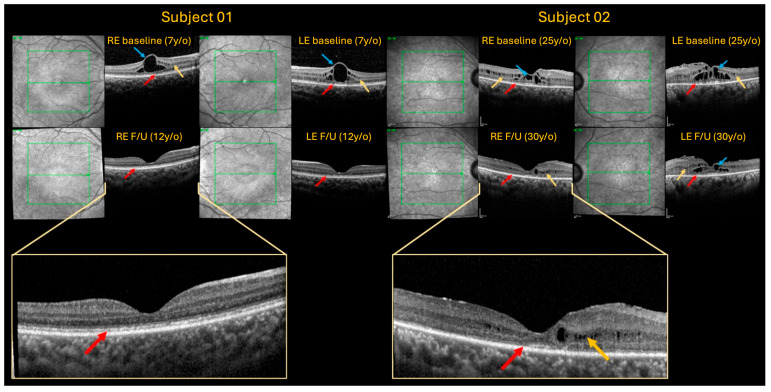
Spectralis OCT imaging of each case. Imaged are ~20° volume scans with the visualised central slices from each eye provided at baseline (**top rows**) and follow up (F/U) (**bottom rows**), for subject 1 (**left**) and subject 2 (**right**). The bottom images show a magnified view of the central macular OCT scan. Subject 1—baseline images show large intraretinal cyst (blue arrow), with schitic/microcystic changes at the inner nuclear layer (INL) (yellow arrow) and photoreceptor disruption (red arrow) in each eye. Follow up-imaging after 5 years shows resolution of all cystic changes, and improvement in photoreceptor layers showing only mild patchy changes in the ellipsoid zone (red arrow). Subject 1—baseline images show larger intraretinal cysts (blue arrows) affecting LE > RE, with similar microcysts/schitic changes within the INL to subject 2 (yellow arrows). There is significant loss of outer segment layers (red arrow) with sparing of a small foveal area observed at baseline and follow-up.

A 25-old female of African heritage (Nigeria) was referred to Moorfields Eye Hospital (London, UK) for bilateral macular oedema and primary angle-closure glaucoma secondary to iris plateau, treated with bilateral laser iridotomy alongside topical Dorzolamide 2% BD and Ganfort once daily. Symptom onset was at an age of 10 years old, when she noted floaters and flashes of light, and was found to have foveoschisis on OCT. No family history of ocular conditions was reported. Upon examination, her BCVA was 0.30 LogMAR with either eye. Goldman applanation tonometry revealed IOP 29 mmHg on either eye (central corneal thickness RE: 528 μm, LE: 549 μm). Slit lamp assessment depicted a shallow anterior chamber confirmed with IOL master 2.21 mm RE and 2.29 mm LE (normal range: 2.5–3.5 mm). Fundoscopy showed fine yellow punctate deposits and blunt macular reflex on either eye; optic nerve showed a C/D of 0.5 on the right eye and 0.7 on the left eye (Figure 3A). FAF confirmed parafoveal hyper-fluorescence and no peripheral FAF abnormalities (Figure 3B). Baseline OCT imaging revealed large intraretinal foveal cysts, subtler microcyst/schitic changes within the OPL, increased central retinal thickness (CRT), central ellipsoid zone disruption with coarse lamination and epiretinal membranes (EPR) on either eye (Figure 4). The patient was subsequently diagnosed with right primary angle-closure and left primary angle-closure glaucoma and suspected macular dystrophy. Whole exome sequencing identified the homozygous c.2506C>A; p.(Pro836Thr) pathogenic variant in the *CRB1* gene.

BCVA on her most recent visit (5-year follow-up) was stable at 0.32 LogMAR with either eye. IOP was stable (RE: 15 mmHg, LE: 17 mmHg) on topical Dorzolamide 2% BD and Ganfort once daily. Most recent follow-up OCT scans depicted improvement of intraretinal cysts and central retinal thickness (CRT) alongside parafoveal disruption of ellipsoid zone (Table 1). Finally, axial length showed 22.19 mm on the right eye and 22.29 mm on the left eye.

**Table 1:** Summary of subject demographics, genetic results, and clinical characteristics of the 2 cases with biallelic pathogenic variants in *CRB1*.

Case	1	2
**Family number**	Z413096	45590
**Gender**	Female	Female
**Ethnicity**	African decent (Sierra Leone)	African decent (Nigeria)
**Age**	07	25
**Age of onset**	07	10
**Phenotype**	MD	MD
**Zygosity**	Homozygous	Homozygous
**Variant cDNAV** **ariant protein**	c.2506C>A p.Pro836Thr	c.2506C>A p.Pro836Thr
**Follow-up**	7-year follow-up	5-year follow-up
**BCVA LogMAR** **Baseline** **Follow-up**	RE: 0.66 LogMAR, LE: 0.54 LogMAR RE: 0.50 LogMAR, LE: 0.60 LogMAR	RE: 0.30 LogMAR, LE: 0.30 LogMAR RE: 0.32 LogMAR, LE: 0.32 LogMAR
**Refractive error**	RE: −2.50/−2.25 × 167 LE: −2.50/−2.00 × 30	RE: −0.75 LE: −0.75
**IOP mmHg** **Baseline** **Follow-up**	RE: 29 mmHg, LE: 44 mmHg RE: 25 mmHg, LE: 25 mmHg	RE: 29 mmHg, LE: 29 mmHg RE: 15 mmHg, LE: 17 mmHg
**IOP treatment**	Brinzolamide BD and Latanoprost once daily	Dorzolamide 2% BD and Ganfort once daily Bilateral laser Iridotomy
**OCT (CRT) (1 mm^3^)** **Baseline** **Follow-up**	RE 448 µm, LE 488 µm RE 117 µm, LE 123 µm	RE 315 µm, LE 374 µm RE 190 µm, LE 258 µm
**EDTs**	Macular dysfunction with mild inner retinal dysfunction affecting rod and cone pathways	Not available

## 3. Discussion

The described cases reveal a novel genotype–phenotype correlation of the c.2506C>A p.(Pro836Thr) variant of the *CRB1* gene with macular dystrophy (MD) and high IOP predisposing the individual to an increased risk of glaucoma. This highlights the importance of early genetic testing and comprehensive multimodal imaging in patients with *CRB1*-related retinopathies, emphasising the interplay between ocular hypertension, macular involvement, and retinal dysfunction.

Biallelic pathogenic variants in the *CRB1* gene result in a diverse range of retinopathies which cause severe retinal degeneration and early-onset visual impairment [12]. Typically, biallelic mutations in the *CRB1* gene are associated with a severe clinical phenotype with progressive vision loss. In *CRB1*-RP, half of the patients meet the World Health Organization’s criteria for low vision (BCVA ≥ 0.3 and <1.00 LogMAR and/or a visual field diameter < 20°) by age 18, and progress to blindness (BCVA 1.3 LogMAR and/or a visual field diameter < 10°) by age 44 [21]. Cohorts predominantly including patients with the *CRB1*-LCA/EOSRD phenotype have reported a mean BCVA of 1.13 LogMAR [13], whereas cohorts primarily including RP patients have documented BCVA values of 0.8 and 0.7 LogMAR [22,23]. Conversely, in a cohort including MD predominantly associated with the in-frame deletion c.498_506del p.(Ile167_Gly169del) variant, the mean BCVA reported was significantly better, at 0.3 LogMAR. This in-frame deletion variant has shown a milder phenotype with relatively slower progression [16], yet no other variant has been identified to show a milder phenotype. In this study, both cases carrying the homozygous c.2506C>A p.(Pro836Thr) variant presented with the MD phenotype and demonstrated relative stability, evidenced by gross ellipsoid zone preservation on SD-OCT and stable BCVA over 5 to 7 years of follow-up. The relative preservation of photoreceptors raises the possibility that the c.2506C>A p.(Pro836Thr) variant may confer a less severe retinal phenotype with more localised retinal degeneration. Unlike the c.498_506del p.(Ile167_Gly169del) variant, which spares CRB1-B and retains partial CRB1 function at the photoreceptor level, the c.2506C>A p.(Pro836Thr) variant affects both CRB1-A and CRB1-B. However, Missense3D analysis [24] predicts minimal impacts on protein structure for this variant, potentially explaining its milder clinical presentation. Interestingly, Rodriguez-Martinez et al. reported that this variant is strongly associated with the MD phenotype when homozygous, but not in compound heterozygous states, contrasting with the in-frame deletion where the allele dictates the phenotype (work to be presented at ARVO 2025). Additionally, this variant is more prevalent in African populations, with an allele frequency of 0.32%, compared to just 0.01% in other ethnicities (gnomAD v4.10), as seen in the two cases presented in this study.

A less common non-retinal association that has been described in *CRB1* retinopathies is an increased risk of glaucoma [25,26]. Although the exact prevalence among *CRB1* retinopathies and underlying mechanism remains unclear, Talib et al. reported glaucoma in 14% of their *CRB1*-RP patients, significantly higher than the previously reported 5.9% to 8.7% [27]. Among these patients, 71% were diagnosed with angle-closure glaucoma [27]. However, no specific variant correlation with glaucoma nor disease mechanisms were reported. Interestingly, Abe et al. described two cases of primary angle-closure glaucoma and cystoid macular oedema in patients with *CRB1* mutations [26] both of which carried the same allele reported in this study. One case involved a 15-year-old Caucasian female with compound heterozygous variants c.2843G>A p.(Cys948Tyr) and c.2506C>A p.(Pro836Thr). This patient had an axial length of 21.07 mm (right eye) and 20.48 mm (left eye) with shallow anterior chamber depths of 2.73 mm and 2.58 mm, respectively. Ultrasound biomicroscopy revealed an anteriorly positioned ciliary body, likely contributing to elevated IOP [26]. Their second case was the patient’s younger brother (8 years old), who also presented with cystoid macular oedema. Gonioscopic examination showed an appositional angle-closure with an IOP of 19 mmHg in both eyes which warranted hypotensive topical medication. Similarly, Sun et al. reported a 7-year-old with *CRB1*-EOSRD presenting with macular oedema, high IOP (RE: 38 mmHg, LE: 35 mmHg), and nanophthalmos (axial length of 20 mm in both eyes). This patient carried missense variants c.1405T > G p.(Cys469Gly) and c.2741G>A p.(Arg905Gln). In this case, the high IOP was attributed to nanophthalmos, characterised by short axial lengths, shallow anterior chambers, scleral thickening, and anomalies in the vein plexus, all of which predispose individuals to angle-closure glaucoma [25]. While axial length and anterior chamber depth measurements were unavailable for case 1 in our study, her myopic prescription suggests an absence of nanophthalmos. However, an anteriorly positioned ciliary body cannot be ruled out as a contributing factor to the elevated IOP. Case 2 had an axial length within normal limits but demonstrated a persistently shallow anterior chamber, even after laser iridotomy, raising suspicion of an anteriorly positioned ciliary body as a potential mechanism.

The electroretinographic features of case 1 show bilateral macular cone dysfunction, with evidence of mild generalised inner retinal dysfunction. Macular cone dysfunction is somewhat expected within the FAF and OCT imaging findings of abnormal macular integrity and PR layer disruption and is consistent with a mild phenotype of *CRB1*-MD [16]. The mild inner retinal dysfunction, evidenced by the low ffERG b:a ratio and subnormal b-wave amplitudes, is less typical. The reduced b-wave features are not specific within the context of this case, and most typically *CRB1* associated MD presents with normal ERGs or perhaps mild cone dysfunction [28,29]. Whilst low b:a ratios can be observed in association with high myopia [30], they can also be associated with a wide range of other conditions such as severe RGC loss, autoimmune/inflammatory disease, or other IRDs [31]. However, this patient was observed to have only mild myopia, with no RNFL thinning, and the mild extent of dysfunction appeared incompatible with an IRD such as complete congenital stationary night blindness. Notably, a review of other genes previously associated with both primary and secondary childhood glaucoma revealed no disease-causing mutations. This included genes such as *CYP1B1*, *PDE6B*, *NDP*, *ATOH7*, *FZD4*, *LRP5*, *TSPAN12*, *HCCS*, *OTX2*, *COL18A1*, *COL11A1*, *BEST1*, and *MFRP* [28,29,30]

Other subtle electroretinographic features were observed. The prolonged On–Off LA ffERG demonstrated a broad ON response and a simplified OFF response, while the DA10 ERG showed slow oscillations following the b-wave. The bifid appearance of the DA 10 b-wave has been scantly documented, though has been reported in association with autoimmune/inflammatory pathology [32]. Whilst the slow oscillations have an uncertain retinal mechanism, we would speculate in the context of abnormal morphology of prolonged On-Off responses, these likely reflect abnormal post-phototransduction signalling within the bipolar cell pathways. Interestingly, a low b:a ratio can also be observed in uveitic disease [33]. Whilst a prolonged LA 3 b-wave peak-time was not observed at the time of testing, the patient had already undergone CMO treatment by this point which may have reversed some of the more widespread inflammatory ERG changes [34]. These electroretinographic features, together and alongside clinical evidence of high IOP and CMO, consummate the evidence of *CRB1*-related disease having a potential inflammatory process, as speculated elsewhere [35,36,37].

The depicted cases underscore the multifaceted role of the *CRB1* gene in eye development beyond the retina, but its complete function is yet to be discovered. As gene therapy for *CRB1*-retinopathies is underway, key aspects to consider before treatment should include evaluating the patient’s natural history, and with that, the therapeutic window. Additionally, identifying potential risk factors such as glaucoma should be noted since it may result in irreversible blindness if left untreated. In cases of *CRB1*-retinopathies where retinal lamination and scaffolding is affected due to abnormal function of both photoreceptors and Muller cells [2], the presence of CMO or lamellar pseudoholes may contraindicate subretinal injection due to the risk of complications like macular hole formation. Similarly, nanophthalmic eyes present challenges, as intraocular surgeries in such cases are associated with higher complication rates, though data on subretinal gene therapy in nanophthalmic eyes is negligible. Proper preoperative assessment and meticulous surgical planning are critical to minimise risks in these high-risk cases. Furthermore, the presence of a narrow anterior chamber angle increases the risk of acute angle-closure glaucoma during gene therapy trials due to repeated mydriasis for dark adapted outcome measures. Thus, biometric analysis and assessment of the anterior chamber angle and intraocular pressure should be routine in patients with *CRB1*-retinopathies, especially for those carrying the c.2506C>A p.(Pro836Thr) variant. For at-risk patients, education on warning symptoms is essential, and prophylactic peripheral iridotomy may be indicated in the contralateral eye to prevent acute angle-closure glaucoma.

## 4. Materials and Methods

Comprehensive ophthalmologic examinations were conducted as part of routine care. Case 1 was seen at Great Ormond Street Hospital (London, UK) where written consent was obtained for publication. Case 2 was identified from the prospectively consented Moorfields Eye Hospital Inherited Eye Disease Database for structure/function of genetic diseases (Research Ethics Number: 12/LO/0141). All procedures adhered to the tenets of the Declaration of Helsinki.

## 5. Conclusions

Our findings identify a novel association between the *CRB1* c.2506C>A p.(Pro836Thr) variant and MD, suggesting a relatively mild and stable phenotype in homozygous cases. Additionally, this variant may predispose to elevated IOP and PACG, further expanding the spectrum of *CRB1*-related ocular manifestations. These observations emphasise the importance of early genetic testing, multimodal imaging, electroretinography, and vigilant monitoring for glaucoma in patients with *CRB1* mutations. Recognising these associations could guide clinical management and improve long-term outcomes for affected individuals.

## Data Availability

The summarised data presented in this study are provided in Table 1. Full datasets are available on request from the corresponding author.
